# Lentiviral Infections Persist in Brain despite Effective Antiretroviral Therapy and Neuroimmune Activation

**DOI:** 10.1128/mBio.02784-21

**Published:** 2021-12-14

**Authors:** Nazanin Mohammadzadeh, Weston Roda, William G. Branton, Julien Clain, Henintsoa Rabezanahary, Ouafa Zghidi-Abouzid, Benjamin B. Gelman, Jonathan B. Angel, Eric A. Cohen, M. John Gill, Michael Li, Jérome Estaquier, Christopher Power

**Affiliations:** a Department of Medical Microbiology and Immunology, University of Alberta, Edmonton, Alberta, Canada; b Mathematical & Statistical Sciences, University of Alberta, Edmonton, Alberta, Canada; c Division of Neurology, Department of Medicine, University of Alberta, Edmonton, Alberta, Canada; d Department of Pathophysiology, CHU de Québec, Université Laval Research Center, Québec, Quebec, Canada; e Department of Pathology, University of Texas Medical Branch, Galveston, Texas, USA; f University of Ottawa and Ottawa Hospital Research Institute, Ottawa, Ontario, Canada; g Institut de Recherches Cliniques de Montréal, Université de Montréal, Montreal, Quebec, Canada; h Department of Microbiology, Infectiology, and Immunology, Université de Montréal, Montreal, Quebec, Canada; i Department of Medicine, University of Calgary, Calgary, Alberta, Canada; McMaster University

**Keywords:** ART, central nervous system infections, human immunodeficiency virus, simian immunodeficiency virus, viral reservoirs

## Abstract

HIV infection persists in different tissue reservoirs among people with HIV (PWH) despite effective antiretroviral therapy (ART). In the brain, lentiviruses replicate principally in microglia and trafficking macrophages. The impact of ART on this viral reservoir is unknown. We investigated the activity of contemporary ART in various models of lentivirus brain infection. HIV-1 RNA and total and integrated DNA were detected in cerebral cortex from all PWH (*n* = 15), regardless of ART duration or concurrent plasma viral quantity and, interestingly, integrated proviral DNA levels in brain were significantly higher in the aviremic ART-treated group (*P *< 0.005). Most ART drugs tested (dolutegravir, ritonavir, raltegravir, and emtricitabine) displayed significantly lower 50% effective concentration (EC_50_) values in lymphocytes than in microglia, except tenofovir, which showed 1.5-fold greater activity in microglia (*P *< 0.05). In SIV-infected Chinese rhesus macaques, despite receiving suppressive (*n* = 7) or interrupted (*n* = 8) ART, brain tissues had similar SIV-encoded RNA and total and integrated DNA levels compared to brains from infected animals without ART (*n* = 3). SIV and HIV-1 capsid antigens were immunodetected in brain, principally in microglia/macrophages, regardless of ART duration and outcome. Antiviral immune responses were comparable in the brains of ART-treated and untreated HIV- and SIV-infected hosts. Both HIV-1 and SIV persist in brain tissues despite contemporary ART, with undetectable virus in blood. ART interruption exerted minimal effect on the SIV brain reservoir and did not alter the neuroimmune response profile. These studies underscore the importance of augmenting ART potency in different tissue compartments.

## INTRODUCTION

All lentiviruses can infect the brain, leading to various severities of neurological disease depending on both the virus and host (1–5). Another common feature of lentiviruses is their capacity to infect cells with macrophage-like phenotypes in the brain (6–8). HIV-1 enters the brain within days after primary infection by infected cells trafficking across the blood-brain barrier (BBB) into the brain parenchyma (6, 9). In the brain, HIV-1 infects resident microglia, trafficking perivascular macrophages, and astrocytes (reviewed in references [Bibr B10][Bibr B11][Bibr B14]). Microglia are long-lived yolk sac-derived CCR5^+^ cells that support HIV-1 productive infection as well as latency (12, 15). HIV-1 infection of the brain induces innate immune responses and nonautonomous cell damage and/or death of neurons (reviewed in references 10, 11 and 16), resulting in neurological disease. HIV-associated neurocognitive disorder (HAND) occurs in people with HIV (PWH), evident as impaired memory, motor functions, and neurobehavioral perturbations (reviewed in references 10 and 11). Current ART decreases viral load in plasma and cerebrospinal fluid (CSF) to undetectable levels (17). With the availability of such ART, the severity of neurocognitive impairments has diminished among PWH, although over 25% may exhibit neurological disabilities, underscoring the limited efficacy of ART (18–21). An active brain viral reservoir might serve as a source of resurgent viral infection after ART interruption or by viruses that have acquired drug resistance mutations due to suboptimal levels of ART drugs (22).

Several studies have reported detectable HIV-1 RNA and DNA in brain tissues from PWH receiving ART (18, 22–25) and SIV-infected nonhuman primates (NHPs) (26–29). In contrast, humanized mice receiving effective ART showed undetectable HIV-1 in the brain (24). Ferrara and colleagues measured concentrations of ART drugs in postmortem brains of HIV-infected persons receiving different ART regimens and compared these results to published concentrations of ART in CSF and found that tenofovir, efavirenz, and lopinavir had higher concentrations in brain compared to CSF, ascribing impaired neurocognitive performance to ART-associated neurotoxicity (30). These circumstances make it imperative to understand the magnitude of the active viral reservoir in the brain and the impact of effective ART on this reservoir in PWH.

There are several obstacles to achieving optimal ART activity in the brain, including low penetration of ART across the BBB with reduced tissue concentrations, physiochemical properties of individual ART drugs (e.g., charge, size, and lipophilicity) precluding their efficacy in the brain, differential expression levels of transporter proteins on brain cells, as well as variable cell type composition, metabolism, and viral replication kinetics (reviewed in reference 31). Moreover, uncertainty regarding ART adherence continues to be a major challenge in the care of PWH that can affect ART activity. ART drug concentrations in serum, liver, and different regions of brain tissue of adult mice at 1 and 4 h after intraperitoneal injection of raltegravir (RAL) and darunavir (DRV) showed markedly lower concentrations of both drugs in brain compared to those in serum and liver (24). Thus, the impact of ART on viral quantities in brain, including viral RNA, integrated proviral and total DNA, as well as viral protein expression, is unclear. The impact of ART interruption of differing durations on viral load in brain remains unknown. Discontinuation or interruption of ART may result in activation of viral reservoirs in different tissues leading to rebound systemic infection, development of drug resistance mutations, and progression of disease. The relative susceptibilities of HIV-infected primary human microglia versus lymphocytes to contemporaneous ART drugs is also unclear. To address these questions, we investigated ART activity in the brain by means of several experimental platforms, including brain tissues from either PWH who had undetectable plasma viral load from ART use for over 5 years on different regimens or those who had uncontrolled plasma viremia at the time of tissue collection. Based on these studies, the effect of a contemporary ART regimen was examined in cultured primary human cells infected by HIV-1 and in a cohort of SIV-infected NHPs. Our working hypothesis was that ART exerted differential effects on viral RNA, integrated proviral and total viral DNA, as well as immune responses in the brain. These studies showed that aviremic PWH receiving suppressive ART displayed HIV-1 genomes and proteins in brain. Moreover, ART and its interruption exerted limited effects on SIV reservoirs and neuroimmune responses in different brain regions, while specific ART drugs affected viral replication differentially in brain microglia compared to lymphocytes.

## RESULTS

### ART effects on brain viral quantities in PWH.

**FIG 1:**
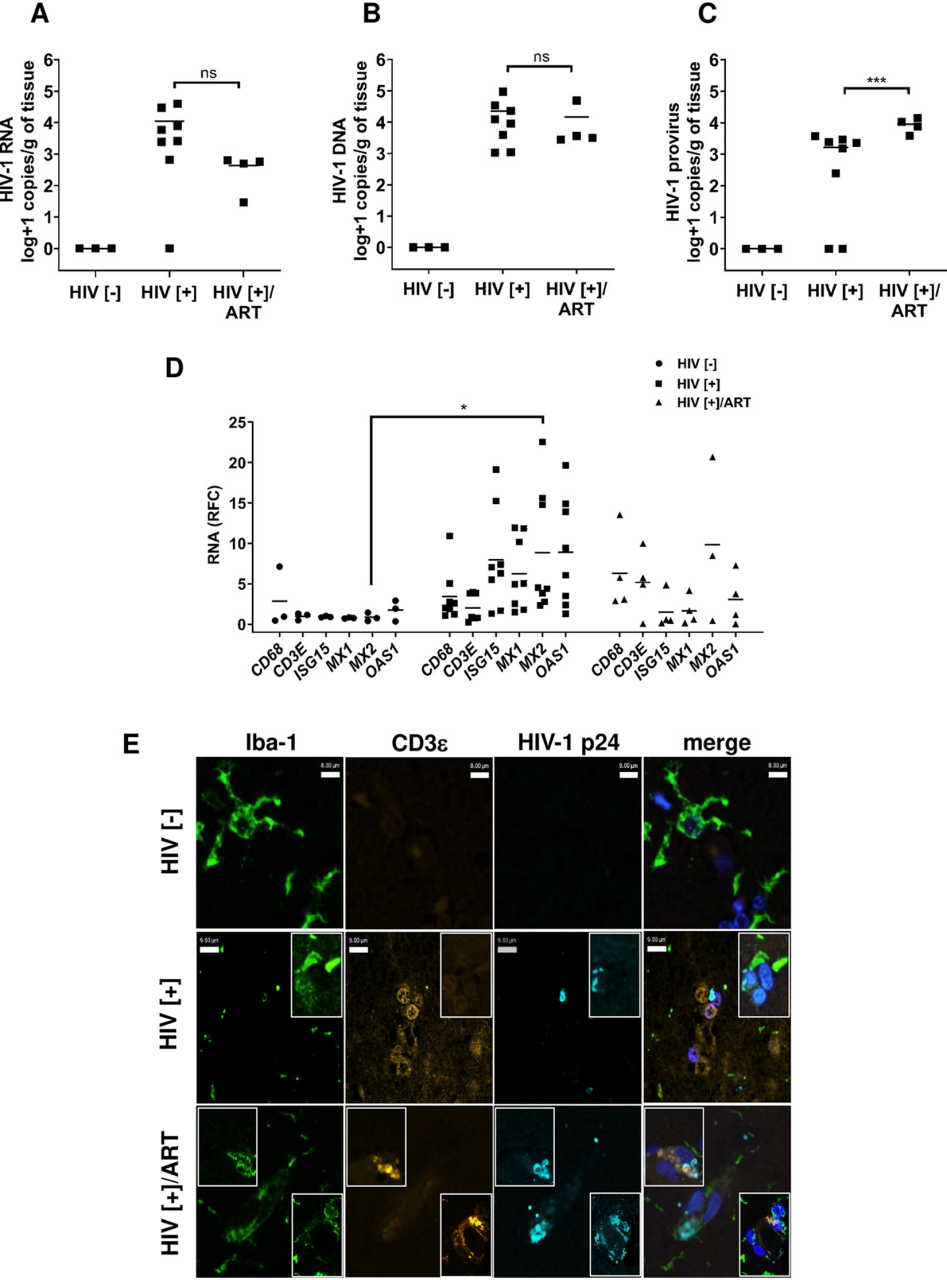
HIV-1 load and host neuroimmune quantitation in human brain tissues. Three experimental groups were examined to determine HIV quantities in brain from 15 persons—uninfected persons (HIV [−], *n* = 3) who died of other causes, HIV-infected persons without plasma viral suppression (HIV[+], *n* = 8), and HIV-infected persons with plasma viral suppression HIV [+]/ART, *n* = 4). (A to C) Based on ddPCR, measurements of (A) HIV-1 RNA, (B) HIV-1 DNA, and (C) HIV-1 provirus (integrated DNA) were assessed. While viral RNA and total DNA levels did not differ between the HIV[+] groups, integrated provirus levels were significantly increased in the HIV[+]/ART group. (D) Host neuroimmune transcript levels in brain were measured by RT-qPCR, including *CD68*, *CD3E*, and interferon-stimulated genes (*ISG15*, *MX1*, *MX2*, and *OAS1*) showing transcript induction in both HIV-infected groups relative to the HIV[–] control group. Only *MX2* showed a significant difference in transcript levels between the HIV[–] and the HIV[+] groups. The horizontal bars in panels A, B, C, and D represent mean values. (E) Immunofluorescence labeling of brain sections from each group showed detection of HIV-1 p24 capsid (cyan) protein in microglia (Iba-1, green) and T-cells (CD3ε, yellow; see the inset) in all HIV[+] groups. Nuclei were labeled with DAPI (4′,6-diamidino-2-phenylindole; blue). (*, *P* < 0.05; **, *P* < 0.01; ***, *P* < 0.001).

The effects of ART on brain viral levels among aviremic PWH is uncertain, prompting examination of viral RNA and total and integrated DNA in cerebral cortex from PWH with uncontrolled plasma virus (HIV[+]), undetectable plasma virus (HIV[+]/ART), and uninfected (HIV[−]) persons (Table 1). Mean viral RNA levels (± standard deviation [SD]) in brain were undetectable in the HIV[−] group (Fig. 1A). The HIV[+] group showed a trend toward higher viral RNA levels (4.0 ± 4.2 log copies/g) compared to the HIV[+]/ART group (2.6 ± 2.4 log copies/g). Analyses of mean total viral DNA levels in brain revealed no viral DNA in control HIV[−] brains as expected. The mean total viral DNA levels in HIV[+] (4.3 ± 4.5 log copies/g) and HIV[+]/ART (4.2 ± 4.4 log copies/g) were similar (Fig. 1B). Mean integrated provirus levels in brain were absent in the HIV[–] group and were significantly higher in HIV[+]/ART group (4.06 ± 3.72 log copies/g), relative to the HIV[+] group (3.2 ± 3.2 log copies/g) (Fig. 1C).

**TABLE 1:** Human cohort of uninfected (HIV[−]) and HIV-infected with viremia (HIV[+]) or without viremia (HIV[+]/ART)

Group	Sex	Mean age (yrs) ± SD	Mean plasma viral load (log copies/ml) ± SD	Mean CD4^+^ T-cell cells/μL ± SD
HIV[–] (*n* = 3)	3 M	42.7 ± 9.6	ND^*a*^	Not available
HIV[+] (*n* = 8)	6 M/2 F	40 ± 6	5.63 ± 5.53	67 ± 113
HIV[+]/ART (*n* = 4)	4 M	57.8 ± 14.2	ND	560 ± 201

a ND, not detectable.

The neuroimmune responses in brain were assessed in each group (HIV[−], HIV[+], and HIV[+]/ART), at the transcript levels for *CD68* (macrophage/microglia marker), *CD3E* (T-cell marker), and selected interferon-stimulated genes (ISGs), including *MX1*, *MX2*, *ISG15*, and *OAS1* (Fig. 1D). *CD68* and *CD3E* mean induction levels were higher in both HIV[+] groups compared to the HIV[−] group. Likewise, mean expression of antiviral interferon-stimulated host restriction factor genes was also higher in both HIV[+] groups with a trend toward lower levels in the HIV[+]/ART group. Notably, *MX2* expression in the HIV[+] group was significantly higher compared to the HIV[−] group. Correlations between viral quantities in brain with host variables (using all available variables) were performed (Fig. S1); the correlation matrix showed that expected plasma viral RNA was negatively associated with blood CD4^+^ T-cell count and positively associated with brain *MX1* and *ISG15* transcript levels. Interestingly, blood CD4^+^ T-cell count was negatively associated with brain *MX1* and *ISG15* expression but positively correlated with brain *CD68* expression. Multivariable regression analysis revealed that HIV-1 RNA levels in brain were not associated with specific host factors, while total HIV-1 DNA levels in brain were positively associated with HIV-1 RNA levels (coefficient, 2.5; *P *< 0.05) but negatively related to brain *CD3E* expression (coefficient, −1,381.0; *P *< 0.05).

10.1128/mBio.02784-21.1 FIG S1 A correlation matrix of human neuroimmune response, brain viral load, age and, plasma CD4^+^ T-cell and viral copies in HIV[+] individuals (Spearman). Correlation coefficients (*r*) appear on the bottom left, and a graphical display of these values appears on the top right. Blue-tinted ellipses represent positive correlations, and red-tinted ellipses represent negative correlations. The boldness of the color and shape of the ellipse represent the strength of the relationship between variables, with stronger correlations having bolder colors and narrower ellipses. All denoted correlations represent a statistical significance of *P *< 0.05. Download FIG S1, TIF file, 0.6 MB. Copyright © 2021 Mohammadzadeh et al. 2021 Mohammadzadeh et al. https://creativecommons.org/licenses/by/4.0/ This content is distributed under the terms of the Creative Commons Attribution 4.0 International license.

Given the long duration of both infection and ART exposure, it was important to determine if viral antigen was present in brains from each group. Brain sections were immunolabeled with antibodies to HIV-1 p24 as well as Iba-1 (microglia/macrophage) and CD3ε (T cell) (Fig. 1E). In sections of frontal lobe from HIV[–] patients, p24 immunoreactivity was absent with little evident CD3ε presence. In contrast, p24 immunoreactivity was apparent in both parenchymal Iba-1 immunopositive microglia/macrophages and perivascular T cells in the HIV[+] and HIV[+]/ART groups (Fig. 1E). From these studies, it was evident that while ART had marked effects on HIV-1 quantity in plasma, its effects on viral quantity in brain were limited, with minimal impact on host neuroimmune responses. As these studies were predicated on different ART regimens, it was important to investigate contemporary ART drugs’ effects in optimized experimental models.

### ART activity in HIV-infected microglia and lymphocytes.

**FIG 2:**
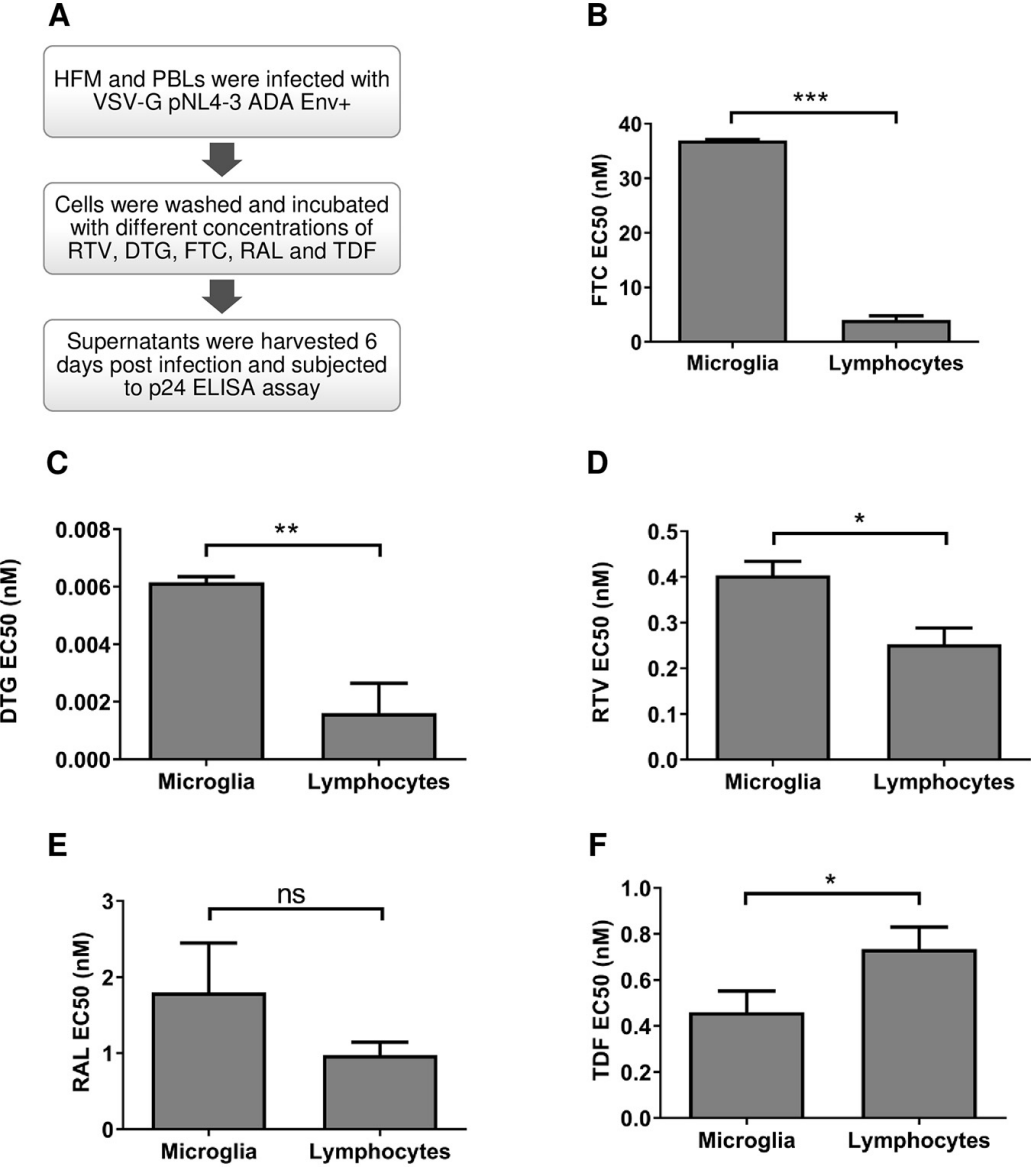
Effect of ART drugs on infected human fetal microglia (HFM) and peripheral blood lymphocytes (PBLs). (A) HFM and PBLs were infected with VSV-G/HIV-1_NL4-3-ADA-Env_^+^ (MOI, 0.02 and 0.01, respectively) and exposed to different concentrations (–3, −2, −1, 0, 1, 2, and 3 log nM) of individual ART drugs—emtricitabine (FTC), ritonavir (RTV), dolutegravir (DTG), raltegravir (RAL), and tenofovir (TDF). (B to F) EC_50_ values for (B) FTC, (C) DTG, (D) RTV, (E) RAL, and (F) TDF were quantified in each infected cell type 6 days postinfection. (Unpaired *t* test; *, *P* < 0.05; **, *P* < 0.01; ***, *P* < 0.001).

Microglia are the most abundant macrophage-like cells in the brain and are permissive to HIV-1 infection (12). To understand the susceptibility of HIV-infected microglia to ART drugs and determine the antiviral activity of ART drugs in human fetal microglia (HFM) versus peripheral blood lymphocytes (PBLs), several drugs in current clinical practice, including tenofovir (TDF), raltegravir (RAL), dolutegravir (DTG), ritonavir (RTV), and emtricitabine (FTC), were investigated (Fig. 2A). All drugs were assessed in a concentration-dependent manner by measuring p24 levels in supernatants from HIV-infected microglia (Fig. S2A) and lymphocytes (Fig. S2B), permitting 50% effective concentration (EC_50_) value calculations. Of note, mean HIV-1 p24 levels in untreated/control lymphocytes and microglia were 31,152 pg/mL and 5,395 pg/mL, respectively. Analyses of ART drugs’ potencies in each infected cell type showed that FTC (Fig. 2B), DTG (Fig. 2C), and RTV (Fig. 2D) displayed significantly higher EC_50_ values in microglia relative to lymphocytes. Following the same trend, RAL (Fig. 2E) had higher EC_50_ values in microglia but showed no significant statistical differences compared to lymphocytes, while TDF inhibited viral production to a greater degree in HIV-infected microglia compared to lymphocytes (Fig. 2F). These studies pointed to more effective antiviral activity of these contemporary drugs in HIV-infected lymphocytes compared to microglia.

10.1128/mBio.02784-21.2 FIG S2 (A and B) Representative figures of the impact of antiretroviral drugs on HIV-1 infection, by measuring HIV-1 p24 in infected (A) HFM and (B) PBLs relative to uninfected controls. Download FIG S2, TIF file, 0.2 MB. Copyright © 2021 Mohammadzadeh et al. 2021 Mohammadzadeh et al. https://creativecommons.org/licenses/by/4.0/ This content is distributed under the terms of the Creative Commons Attribution 4.0 International license.

### ART effects on brain SIV burden.

**FIG 3:**
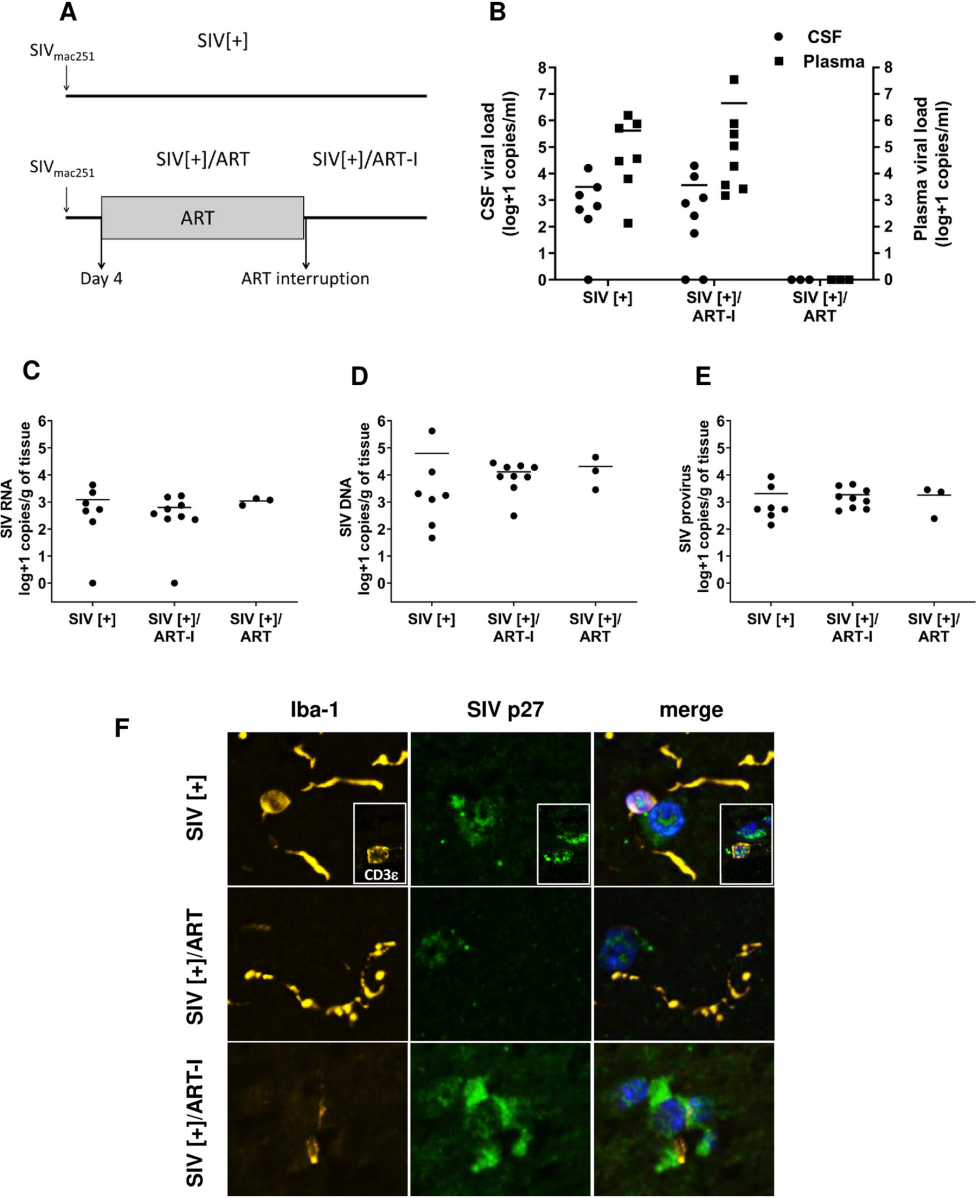
Comparison of SIV quantities in plasma, CSF, and brain tissues from nonhuman primates. (A) Three experimental groups of Chinese rhesus macaques were investigated to determine SIV load in different compartments (plasma, CSF, and brain) after infection. All animals were infected with SIV_mac251_ (10 50% animal infectious dose [AID_50_]). SIV-infected animals (SIV[+], *n* = 7) did not receive ART after infection, and animals were euthanized 38 to 104 days postinfection. The SIV[+] group in which ART was interrupted (SIV[+]/ART-I, *n* = 8) received a combined regimen of RAL/DTG, FTC, RTV, and TFV, beginning 4 days after primary infection, for 56 days to ensure undetectable plasma viral load. After SIV suppression, therapy was stopped, and animals were euthanized when viral RNA was detectable in plasma; ART interruption duration varied from 10 to 28 days, with two animals requiring 159 and 161 days to rebound. The SIV-treated group without interruption (SIV[+]/ART, *n* = 3) had suppressed plasma viral load after receiving a combined regimen of RAL/DTG, FTC, RTV, and TFV, also beginning 4 days postinfection, for the duration of 27 to 55 days until they had undetectable plasma viral load and were euthanized at that time. (B) Plasma and CSF viral RNA levels from the three experimental groups were measured at the time of death. (C to E) Total RNA and DNA were prepared from postmortem brain tissues from all animals and subjected to ddPCR for the measurement of SIV (C) RNA, (D) total DNA, and (E) provirus (integrated DNA). The horizontal bars in panels B, C, D, and E represent mean values. (F) Immunofluorescence microscopy of brain sections from each experimental group displayed SIV p27 (capsid, green) immunoreactivity in microglia (Iba-1, yellow) and T cells (CD3ε, yellow; see the inset) in all three groups with DAPI nuclear staining (blue).

**FIG 4:**
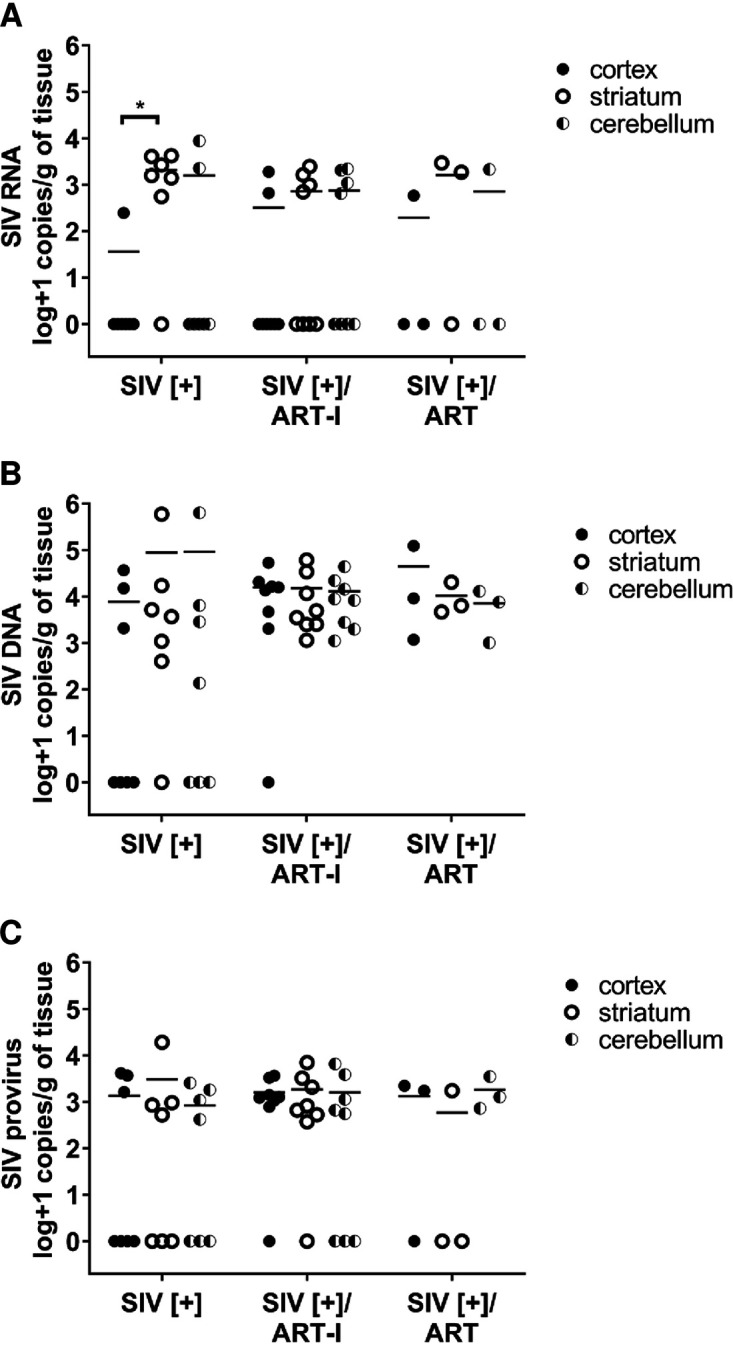
SIV burden in three different brain regions of Chinese rhesus macaques. (A) SIV RNA, (B) SIV DNA, and (C) SIV provirus (integrated DNA) in cortex, striatum, and cerebellum.

Many studies have examined the *in vivo* impact of ART on different viral variables within brain in SIV infection models (27, 32, 33) as well as multiple HIV-1 infection models, including humanized mice (34, 35). Nonetheless, the effects of early introduction of ART after primary infection and its subsequent interruption on brain viral load, especially on integrated provirus levels, remained uncertain. We examined the effects of the same ART drugs assessed above in the present SIV model that included three groups: (i) SIV infection without ART (SIV[+]), (ii) SIV infection with ART interruption after 56 days of therapy (SIV[+]/ART-I), and (iii) SIV infection with sustained and suppressive therapy for 27 to 55 days (SIV[+]/ART) (Table 2). ART initiation occurred within 4 days of primary infection. For the SIV[+]/ART-I group, ART was interrupted, and the study was terminated upon animals becoming viremic (Fig. 3A). Mean SIV RNA levels in plasma at the time of experiment termination ranged widely among the SIV[+] (5.6 ± 5.7 log copies/mL) and the SIV[+]/ART-I groups (6.6 ± 7.0 log copies/mL) but was not detected in plasma from the SIV[+]/ART group (Fig. 3B). Similarly, mean viral RNA levels in CSF were undetectable in the SIV[+]/ART group, while viral RNA was detectable in the SIV[+] (3.4 ± 3.7 log copies/mL) and the SIV[+]/ART-I groups (3.5 ± 3.8 log copies/mL) at the end of the study (Fig. 3B). In the brain tissues from the same groups, viral RNA or DNA quantities did not differ based on comparisons of frontal cortex, striatum, or cerebellum, although viral RNA in the SIV[+] group was significantly lower in cortex compared to striatum (Fig. 4A). Neuropathological studies showed minimal gliosis in white and gray matter among all experimental groups without evidence of opportunistic infections or pathological lesions such as microglial nodules and/or multinucleated giant cells. A test of independence showed that there was no association between the three anatomic regions and SIV quantity (Chi-square, 5.855; *P *= 0.0535) (Fig. 4A to C). Thus, data from each anatomic site were averaged within each animal. Mean viral RNA levels in brain showed minimal variability among groups—SIV[+] (3.09 ± 3.19 log copies/g), SIV[+]/ART-I (2.78 + 3.21 log copies/g), and SIV[+]/ART (2.92 ± 3.19 log copies/g) (Fig. 3C). Likewise, mean total viral DNA levels in brain displayed little variation between groups—SIV[+] (4.80 ± 5.20 log copies/g), SIV[+]/ART-I (4.17 ± 3.69 log copies/g), and SIV[+]/ART (4.32 ± 4.04 log copies/g) (Fig. 3D). Measurement of integrated DNA levels in brain revealed minimal differences among groups—SIV[+] (3.24 ± 3.49 log copies/g), SIV[+]/ART-I (3.23 ± 2.68 log copies/g), and SIV[+]/ART (3.10 ± 3.17 log copies/g) (Fig. 3E).

**TABLE 2:** SIV-infected nonhuman primate cohort of SIV-infected (SIV[+]), ART interrupted (SIV[+]/ART-I) or receiving suppressive ART (SIV[+]/ART)^*a*^

Group	Sex	Mean age (yrs) ± SD	Mean plasma viral load (log copies/ml) ± SD	Mean CSF viral load (log copies/ml) ± SD	Mean CD3^+^ CD4^+^ (%) ± SD
SIV[+] (*n* = 7)	F	6.4 ± 1.2	5.62 ± 5.77	3.49 ± 3.76	30 ± 6
SIV[+] /ART-I (*n* = 8)	F	5.2 ± 0.4	6.65 ± 7.09	3.57 ± 3.86	59 ± 10
SIV[+]/ART (*n* = 3)	F	5.8 ± 1.0	ND	ND	49 ± 6

a ND, not detectable.

As viral RNA and DNA were detected in brains from all animals, the presence of viral antigen in brain from each group was assessed. SIV capsid p27 immunofluorescence was colocalized with Iba-1 and CD3ε as described in the human cohort (Fig. 3F). p27 immunoreactivity was evident in parenchyma microglia (Fig. 3F, yellow) and perivascular T-cells (Fig. 3F, inset yellow). Thus, ART implementation and its interruption exerted substantial effects on plasma and CSF viral RNA levels, although there were negligible effects on brain viral RNA, DNA, or protein.

### Neuroimmune response correlation with SIV and host variables.

**FIG 5:**
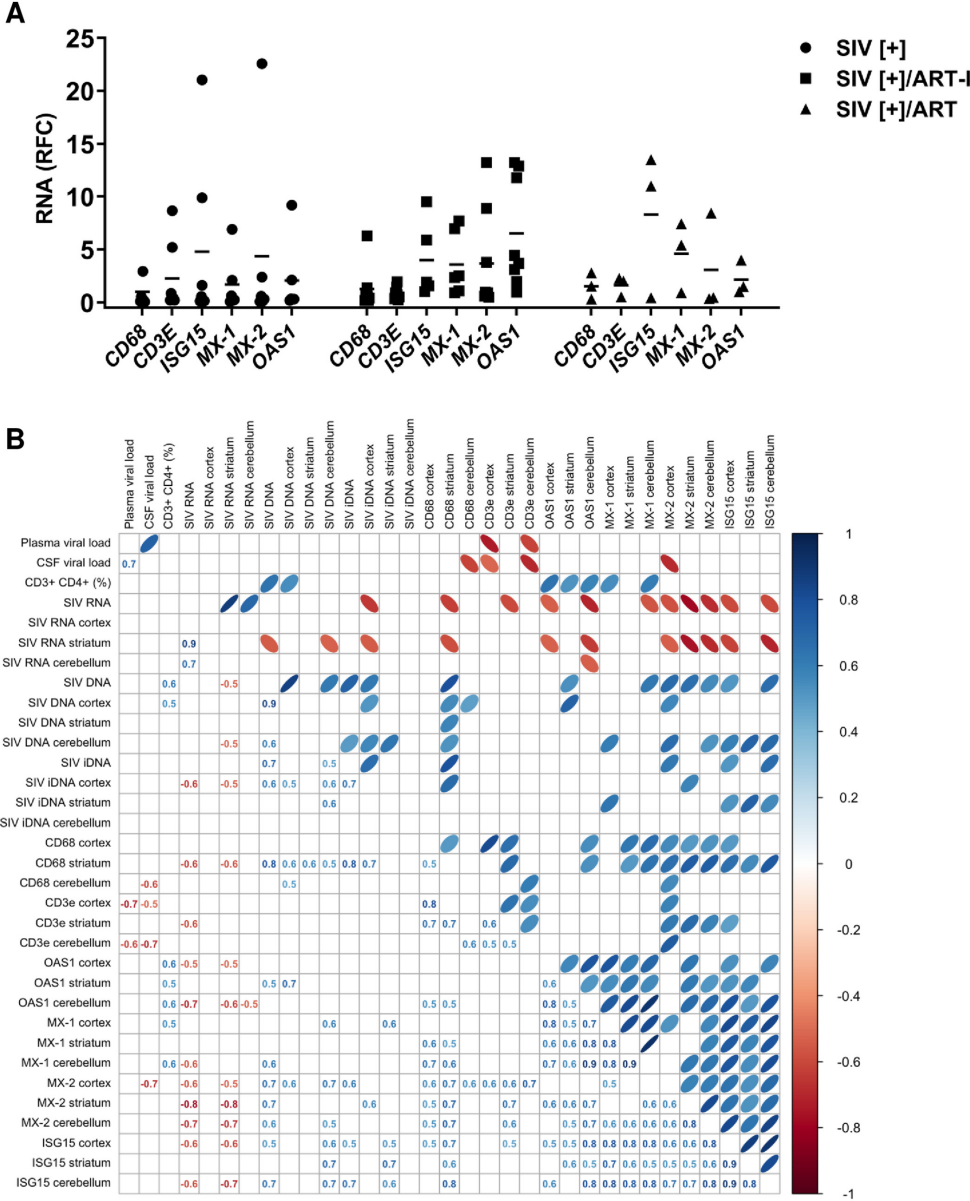
Host neuroimmune responses and correlation analyses in SIV-infected nonhuman primates. (A) Analyses of CD68, CD3E, and interferon-stimulated genes (*ISG15*, *MX-1*, *MX-2*, and *OAS1*) transcript levels showed mean expression for each gene in cortex, striatum, and cerebellum relative to the SIV[+] ART group without significant differences in individual transcript levels between groups. The horizontal bars represent mean values. (B) A correlation matrix of host neuroimmune response and brain viral load in three different regions (cortex, striatum, and cerebellum), as well as ART and plasma/CSF viral load variables. Correlation coefficients (*r*) appear in the left lower matrix, and a graphic display of these values appears on the top upper matrix. Blue-tinted ellipses represent positive correlations, while red-tinted ellipses represent negative correlations. The boldness of the color and shape of the ellipse represent the strength of the relationship between variables, with stronger correlations having bolder colors and narrower ellipses. All denoted correlations represent a statistical significance of *P* < 0.05.

Given the comparative lack of ART-mediated effects on brain viral quantities, the host immune responses in brain were examined in each of the three SIV[+] groups (SIV[+], SIV[+]/ART-I, and SIV[+]/ART) by measuring representative genes’ transcript levels in brain tissues (Fig. 5A). *CD68* and *CD3E* levels were low among all groups with mean induction levels ranging from 1- to 2-fold within the three experimental groups. In contrast, (antiviral) interferon-stimulated and host restriction factor mean induction levels varied widely across all groups, and a distinctive trend was not identified. To investigate the relationships between viral quantities in plasma, CSF, and brain with host neuroimmune responses (in the three different brain regions) as well as other variables (e.g., blood CD4 T-cell percentage), correlational analyses were performed using all available variables (Fig. 5B). These studies showed that host immune gene transcript levels were significantly and positively correlated with each other regardless of anatomic site. In contrast, mean total and striatal viral RNA levels were inversely correlated with host immune transcript abundance, while global total SIV DNA and total SIV DNA in both cerebellum and cortex were positively associated with *CD68* expression and host antiviral responses. Notably, multivariable regression analyses of the SIV-related factors showed that mean integrated proviral DNA levels (coefficient, 525.9; *P *< 0.05) were positively associated with *CD68* levels (coefficient, 525.9; *P *< 0.05). Thus, while ART exerted marked effects on viral quantity in plasma, its effects in the brain in terms of both viral quantity and host immune responses were negligible.

## DISCUSSION

To our knowledge, this is the first study to compare lentiviral RNA and integrated and total viral DNA copy numbers in brain tissues from both SIV-infected NHPs and PWH in the presence, absence, or interruption of effective ART. These data indicated that viral RNA and proviral and total viral DNA levels were similar in brain tissues across experimental groups despite ART-mediated suppression of plasma and CSF viral RNA. Introduction of ART soon after primary infection (4 days) did not prevent SIV neuroinvasion. Moreover, ART interruption exerted minimal effects on brain viral burden in the SIV model. Correlational analyses pointed to antiviral host immune responses in brain tissues from both humans and NHPs as being closely associated components of infection in these models that were unaffected by ART. Immunodetection of viral antigens was evident in both parenchymal and perivascular brain regions, principally in macrophage-like cells, among PWH and SIV-infected animals, regardless of ART exposure. These studies were complemented by observations indicating that some ART drugs have substantially lower activity against HIV-1 infection of microglia relative to lymphocytes. From these studies, it was apparent that brain viral quantities showed limited variation despite effective ART, implying that new therapeutic strategies are required to eradicate HIV-1 from this organ reservoir.

We evaluated SIV RNA, DNA, and integrated DNA in brain and SIV DNA and RNA in plasma and CSF (respectively) from Chinese rhesus macaques receiving effective ART, interrupted ART, or no ART treatment using contemporary antiretroviral drugs. The SIV-infected Chinese rhesus macaque model represents a contemporary platform for studying viral reservoirs in the brain because these animals typically display lower viral load and rarely develop encephalitis, which is also now infrequently observed among PWH who receive suppressive ART. Previous reports found CSF and plasma viral quantities in SIV-infected Chinese rhesus macaques similar to those observed for HIV-1 infection of the brain (27). We also observed that when human peripheral blood lymphocytes and microglia were exposed to different concentrations of contemporary antiretroviral drugs after HIV-1 infection *in vitro*, the EC_50_ for FTC, DTG, and RTV were significantly higher in microglia compared to lymphocytes. The same trend was observed for RAL, although it lacked statistical significance. FTC was the least effective drug in microglia. Conversely, TDF was 1.5 times more efficient in microglia compared to lymphocytes. The striking lower activity of all but one ART drug tested in microglia might be attributable to differential expression of efflux transporter genes in microglia and perhaps pharmacokinetic properties of individual ART drugs in each cell type (31). The same ART drugs were tested in the present SIV-infected model, which suppress plasma and CSF viral quantities to undetectable levels in the continuously treated group. Evaluation of SIV RNA and integrated proviral and total viral DNA in cerebellum, cerebral cortex, and striatum of infected animals showed that brain viral RNA and integrated proviral copy numbers per gram of tissue were similar (except for SIV RNA in the SIV[+] group, which was lower in cortex compared to the striatum), albeit generally a log lower than total viral DNA copy numbers per gram. Immunofluorescence labeling of the SIV capsid protein p27 detected this protein in Iba-1 and CD3ε immunopositive cells in postmortem brain tissues of SIV-infected animals regardless of ART status. These observations were largely reproduced in the human brain samples, with several differences; viral copy numbers for RNA and provirus and total viral DNA were lower in the SIV model, likely reflecting intrinsic features of this model, as well as the longer durations of HIV-1 infection among PWH (all were AIDS-defined). Nonetheless, unlike the SIV model, there was a trend toward reduced viral RNA copy numbers in brains from PWH receiving effective ART at the time of tissue harvest. This modest reduction in viral RNA quantity could be attributable to sustained ART exposure, perhaps also indicating the apparent diminished neuroimmune responses in the same group.

Immunofluorescence detected HIV-1 and SIV capsid proteins (p24 and p27, respectively) in Iba-1 and CD3ε immunopositive cells in the postmortem brain tissues of patients and animals regardless of virus suppression status. However, the animals (and humans) used in this study were not perfused prior to necropsy, raising the possibility of residual blood-associated virus in the intravascular space. Several findings counter this concern, including the similarity in brain viral RNA copy numbers across all three groups’ brains despite substantial differences in blood (and CSF) RNA copy numbers. This point is supported by a lack of anatomical distinction in viral copy numbers, although the striatum and cerebellum contain less vascularity than the frontal cortex. Moreover, immunofluorescence imaging detected viral capsid within the brain parenchyma as well as in perivascular locations but not in the luminal space of blood vessels. Of note, we previously demonstrated the absence of cell-associated SIV DNA in both monocytes and lymphocytes in the blood of the same monkeys treated with early ART (36).

In agreement with our data described above, previous studies have reported that PWH and SIV-infected NHPs have detectable viral DNA in brain tissues despite receiving suppressive ART (27, 32). Lamers et al. detected HIV DNA in various anatomical sites in ART-treated patients with aviremia while on ART (23, 24). Among the current PWH with undetectable plasma HIV-1 at the time of tissue collection, all had been closely followed in established HIV clinical programs, were aviremic for at least 5 years, and had received ART within 12 h of tissue collection. In contrast, PWH with viremia (HIV[+] group) in the present study had been exposed to ART in the past, which prompted investigation of an optimized SIV model. In SIV-infected Chinese rhesus macaques, the levels of SIV gag-encoding DNA were similar in brains of ART-treated and untreated animals, although ART reduced viral load in gray matter more than white matter (27), which was also apparent in the present study in that viral RNA was more often undetectable in cortical samples from SIV-infected NHPs (Fig. 4A). In the present study, there was an enhanced neuroimmune response in the PWH with unsuppressed plasma virus compared to the uninfected control group, and interferon-stimulated genes were positively correlated with brain integrated proviral DNA copy numbers. Indeed, this contemporary ART regimen with differing exposure durations in the NHP cohort coupled with heterogeneous ART composition and duration in the PWH indicated that ART had very limited influence on brain viral quantities, recapitulating earlier studies (18, 25, 37). While it is widely acknowledged that ART reduces the severity of neurocognitive impairments among PWH, the present findings imply that ART’s benefits might be mediated through its systemic effects, including diminished inflammation in blood with effects on the BBB and related functions (38–40). In fact, the BBB represents a major uncertainty in terms of its effects on ART penetrance in CNS tissues as reviewed in reference 41. The existing published data are variable, with experimental studies showing very limited and differential brain penetrance of ART drugs depending on the specific drug and the model system (24, 42, 43). In contrast, a clinical study that analyzed ART drug concentrations in autopsied brain tissues from ART-treated PWH revealed remarkably high drug levels (e.g., tenofovir, efavirenz, and lopinavir) in brain tissues (30), although these data await verification in other studies. Of note, the BBB is a physiological barrier composed of tightly fused brain capillary endothelial cells that restrict the penetration of high-molecular-weight and/or hydrophilic molecules (reviewed in reference 31). Infection by HIV-1 induces damage to the integrity of the BBB, and ART drugs do not ameliorate or enhance this disruption (44, 45). On the other hand, it has been shown that chronic inflammation leads to the upregulation of permeability-glycoprotein (p-gp), which reduces BBB permeability and might lead to diminished ART concentrations in the brain (46). Nonetheless, the situation is likely more complex, as we previously showed that extracellular concentrations of ART drugs were higher in microglia compared to peripheral blood mononuclear cells (PBMCs), and intracellular concentrations of these drugs were higher in PBMCs compared to the microglia (24). We are currently investigating the ART impact on viral burden in microglia, including analyses of drug efflux genes. The effects of BBB perturbation coupled with infected cell type differential susceptibility to ART can exert profound effects on ART drug concentrations and efficacy in brain tissue with ensuing effects on HIV-1 DNA, RNA, and protein levels. Thus, the limited impact of ART on brain HIV-1 or SIV load is a multidimensional and dynamic problem that awaits further analyses.

The present study faced several challenges that included uncertainty regarding ART drug concentration in brain tissue. Moreover, the present ART regimen did not contain the antiretroviral CCR5 antagonist maraviroc, which is reported to improve neurocognitive performance in PWH with HAND (47) and might be more effective in the brain because of the CCR5 dependence for brain-derived HIV-1 and SIV strains (48). The absence of an uninfected NHP group precluded comparisons of host immune responses between infected and uninfected animals, which was obviated in the human cohort by including an uninfected group. These experimental circumstances were driven by institutional ethics guidelines regarding euthanizing uninfected (healthy) animals. In addition, the present SIV study included animals with short durations of infection and treatment compared to the PWH examined in these studies. Finally, the comparative rarity of obtaining brain tissues from patients receiving ART at the time of tissue collection is a limiting factor to drawing broader conclusions about the impact of ART on the brain’s viral quantities among PWH. Nonetheless, it was reassuring that the current findings were comparable in HIV- and SIV-infected brains in terms of both viral quantities and host neuroimmune responses.

The presence and persistence of high SIV RNA and DNA copy numbers in the brains of macaques receiving ART at day 4 postinfection highlights the importance of preexposure therapeutic intervention. Remarkably, ART interruption did not increase SIV RNA or DNA quantities or influence neuroimmune responses in the brain, indicating that brief interruptions in ART may not exert immediate effects on brain viral load, although the emergence of ART resistance variants in brain and in other organs could be a long-term consequence. Given that microglia harbor both latent and active virus that will persist during effective ART in aviremic patients, their contribution to rebound infection and development of drug-resistant variants require further investigation.

## MATERIALS AND METHODS

### Ethics statement.

The use of autopsied human brain tissues was approved (Pro0002291) by the University of Alberta Human Research Ethics Board (Biomedical), and written informed consent was received for all samples. In addition, brain tissue from patients was obtained from the National NeuroAIDS Tissue Consortium (NNTC) collection. Human fetal brain tissues were obtained from 15- to 22-week aborted fetuses that were collected with the written informed consent from the donor (Pro00027660), approved by the University of Alberta Human Research Ethics Board (Biomedical). Chinese rhesus macaques (Macaca mulatta) were housed at Université Laval in accordance with the rules and regulations of the Canadian Council on Animal Care (http://www.ccac.ca). The present study protocol was approved by the Laval University Animal Protection Committee (project number 106004).

### Animal housing and care.

Animals were fed standard monkey chow diet, supplemented daily with fruit and vegetables and water *ad libitum*. Social enrichment was delivered and overseen by veterinary staff, and overall animal health was monitored daily. Animals were evaluated clinically and were humanely euthanized using an overdose of barbiturates according to the guidelines of the Veterinary Medical Association.

### Animal infection and sample collection.

Eighteen rhesus macaques that were seronegative for SIV, simian T leukemia virus type 1 (STLV-1), simian type D retrovirus 1 (SRV-1), and herpes B viruses were infected intravenously with SIVmac251 (10 AID50). At day 4 postinfection, 11 animals were treated daily with tenofovir (TFV, 20 mg/kg; Gilead) and emtricitabine (FTC, 40 mg/kg; Gilead) subcutaneously and raltegravir (RAL, 20 mg/kg; Merck) or dolutegravir (DTG, 5 mg/kg; ViiV) and ritonavir (RTV, 20 mg/kg; Abbvie) orally, as previously described (36). Animals were sacrificed at different time points postinfection (SIV, *n* = 7), receiving ART for the duration of the study (SIV/ART, *n* = 3), and after ART interruption (SIV/ART-I, *n* = 8). Necropsy, including harvest of plasma, cerebrospinal fluid and brain was performed immediately after sacrifice (36).

### RNA and DNA preparation.

Total RNA and DNA were extracted from brains of HIV-infected and uninfected patients (midfrontal gyrus) and SIV-infected animals (parietal cortex, striatum, and cerebellum), as previously described (32, 49), using the RNeasy and DNeasy kits (Qiagen, Germantown MD, USA) according to the manufacturer’s protocol.

### Quantitative real-time reverse transcriptase PCR (RT-PCR).

The cDNA was synthetized using DNase-treated RNA, random primers and Superscript III, and reverse transcriptase (Invitrogen, Carlsbad, CA, USA) at 44°C for 90 min according to the manufacturer’s instructions. cDNA was diluted 1:3 (adding 100 μL of ultrapure water to 50 μL of cDNA). Human and macaque immune genes were quantified and normalized to GAPDH and the appropriate control group using verified primers (Table S1) as previously described (49).

10.1128/mBio.02784-21.5 TABLE S1 Oligonucleotide primers used for quantitative real-time PCR. Download Table S1, DOCX file, 0.02 MB. Copyright © 2021 Mohammadzadeh et al. 2021 Mohammadzadeh et al. https://creativecommons.org/licenses/by/4.0/ This content is distributed under the terms of the Creative Commons Attribution 4.0 International license.

### Droplet digital PCR analyses of viral RNA and total and integrated DNA.

HIV-1 and SIV viral RNA were quantified using 5 μL of diluted cDNA as the template. For the quantification of total viral DNA, 300 ng of genomic DNA (gDNA) was used. For both HIV-1 RNA and total DNA quantification, HIV-1 pol-F and HIV-1 pol-R primers were used, and for both SIV RNA and total DNA quantification, SIV-F and SIV-R primers were used (Table S2). SIV and HIV-1 integrated DNA were quantified using 300 ng of gDNA and human Alu-F and HIV-1 gag-R primer set and macaque Alu-F and SIV gag-R primer set (Table S2). The Bio-Rad QX200 droplet digital PCR (ddPCR) system and QX200 ddPCR EvaGreen supermix were used for quantification as per the manufacturer’s protocol as previously described (50, 51). Droplets were generated from each reaction with a final volume of 20 μL using a Bio-Rad QX200 droplet generator. PCR was performed using an S1000 thermal cycler (Bio-Rad) using the following program: 10 min at 95°C, 30 sec denaturation at 94°C, 58°C extension for 60 sec, and 10 min at 98°C for a total of 40 cycles. After the cycling, droplets were analyzed using a QX200 droplet reader. Raw data were analyzed with the specific software (QX200) by setting a common fixed fluorescence threshold intensity based on the nontemplate control. The number of template copies was then estimated based on the number of positives detected in a corresponding channel and the number of total accepted droplets. Samples were quantified at least in duplicate. Template copies per sample were calculated by averaging the overall replicate wells per sample. The values are reported as copies per gram of tissue with approximately 30 mg of tissue used for initial RNA and DNA extraction.

10.1128/mBio.02784-21.6 TABLE S2 Oligonucleotide primers for ddPCR. Download Table S2, DOCX file, 0.01 MB. Copyright © 2021 Mohammadzadeh et al. 2021 Mohammadzadeh et al. https://creativecommons.org/licenses/by/4.0/ This content is distributed under the terms of the Creative Commons Attribution 4.0 International license.

### Cell cultures.

For *in vitro* ART studies, primary human fetal microglia (HFM) and adult peripheral blood lymphocytes (PBLs) were infected and allowed to replicate in the presence or absence of ART drugs (24, 52). Primary HFM were isolated from fetal brain tissues with a gestational age of 16 to 21 weeks, as previously described (52). The cells were washed twice and plated in vented T-75 tissue culture flasks (Sarstedt), and cultures were incubated at 5% CO_2_ for 2 weeks. Cultures were maintained in minimal essential medium (MEM) supplemented with 10% fetal bovine serum (FBS), 2 mM l-glutamine, 1 mM sodium pyruvate, 1× MEM nonessential amino acids, 0.1% dextrose, 100 U/mL penicillin, and 100 μg/mL streptomycin. After 2 weeks, cultures were gently rocked for 10 min to suspend the weakly adhering microglia in medium, which were then decanted, washed, and plated. The purity of cultures was verified by immunofluorescence (52).

PBLs were isolated from PBMCs of healthy donors by incubating the cells in T-75 tissue culture flasks, allowing adherence of the monocytes to the flask, and transferring the suspended cells containing lymphocytes to the new flask. This was repeated 0.5, 2, and 4 h post-initial seeding. The purity of cultures was verified by immunofluorescence. PBMCs were isolated from whole-blood samples of healthy donors using the Ficoll-Paque (Sigma) density gradient centrifugation and SepMate-50 tubes (STEMCELL Technologies) as per the manufacturer’s protocol. Cells were cultured in RPMI 1640 medium (HyClone) with 10% heat-inactivated FBS (Gibco) and 1% penicillin/streptomycin (HyClone). Cells were stimulated for 3 days at 37°C with 10 ng/mL recombinant human interleukin-2 (IL-2; Sigma) and 10 μg/mL phytohemagglutinin-p (PHA-p) (Roche). Cells were resuspended in RPMI 1640 supplemented with 10% heat-inactivated FBS, 1% penicillin/streptomycin, 1% glutamine, and 1% recombinant human IL-2, at which time cells were infected (24).

### Production of viral stocks.

HIV-1 was produced by cotransfection of HIV-1_NL4-3-ADA-Env_^+^ and a vesicular stomatitis virus G (VSV-G)-encoding plasmid into 1 × 10^6^ 293T cells cultured in Dulbecco’s modified Eagle’s medium (DMEM) with 10% FBS in a 10-cm tissue culture dish (Thermo Fisher). Lipofectamine 3000 (Invitrogen, Carlsbad, CA) was used as the transfection reagent according to the manufacturer’s protocol. The medium was changed 6 h posttransfection, and at 72 h posttransfection, virus was harvested, cleared by centrifugation at 500 × *g* for 10 min, and then frozen at −80°C. HIV-1 p24 was measured by enzyme-linked immunosorbant assay (ELISA), and the titer of the virus was determined using the TZM-bl reporter cell line and the Reed and Muench method to determine the 50% tissue culture infective dose (TCID_50_).

### ART EC_50_ assays in cell cultures.

For *in vitro* ART studies, as previously described (24), HFM were seeded and, the following day, infected with VSV-G/HIV-1_NL4-3-ADA-Env_^+^ at a multiplicity of infection (MOI) 0.02 for 4 h. cells were washed 3 times with phosphate-buffered saline (PBS), and 200 μL of diluted drugs at different concentrations were added to each well postinfection and incubated for 6 days. On day 6 postinfection, supernatants were collected for HIV-1 p24 quantification by HIV-1 p24 antigen capture ELISA (ABL, Inc.).

For PBL infections, as previously described (24), cells were infected with VSV-G/HIV-1_NL4-3-ADA-Env_^+^ for 2 h (MOI, 0.01), washed, and resuspended with fresh medium. Diluted drugs (150 μL) at different concentrations were added to each well. On day 6 postinfection supernatant was collected for HIV-1 p24 quantification. RAL, RTV, FTC, DTG, and TDF were obtained from the NIH AIDS Research and Reference Reagent Program. The EC_50_ levels were determined using GraphPad Prism version 5.

### Immunofluorescence imaging.

Fixed tissues from uninfected and HIV- or SIV-infected brains were paraffin-embedded, followed by sectioning and mounting. Slides were rehydrated and subjected to antigen retrieval by boiling in 10 mM sodium citrate, pH 6, before immunostaining. Tissues were labeled with primary antibodies (anti-Iba-1 [Abcam], anti-hCD3ε [Millipore], anti-HIV-1 p24 and anti-SIV p27] overnight at 4°C. Autoflourescence was quenched using a TrueBlack lipofuscin autofluorescence quencher (Biotium), followed by application of appropriate secondary antibodies (53, 54). Images were acquired on a Wave Fx (Quorum Technologies) spinning disk confocal microscope using Volocity (Perkin Elmer) acquisition and analysis software.

### Statistical analyses.

An unpaired *t test* was used for *in vitro* comparison of ART drug effects. One-way or two-way analysis of variance (ANOVA; where appropriate) and Tukey’s multiple-comparison test were used for the analysis of brain viral load and neuroimmune responses. Statistical tests were applied using Prism version 5 (GraphPad Software, San Diego, California, USA). Spearman correlations were generated using R package corrplot project. Results were expressed as the mean ± SD. *P* values of <0.05 were considered significant. Multivariable regression analyses and locally weighted scatterplot smoothing (LOWESS) were also applied to the data sets (Fig. S3 and S4).

10.1128/mBio.02784-21.3 FIG S3 HIV scatterplot with LOWESS. LOWESS (locally weighted scatterplot smoothing) was used to visually assess the relationship between variable pairs in HIV[+] individuals. Download FIG S3, TIF file, 1.0 MB. Copyright © 2021 Mohammadzadeh et al. 2021 Mohammadzadeh et al. https://creativecommons.org/licenses/by/4.0/ This content is distributed under the terms of the Creative Commons Attribution 4.0 International license.

10.1128/mBio.02784-21.4 FIG S4 SIV scatterplot with LOWESS. LOWESS was used to visually assess the relationship between variable pairs in the NHP cohort. Download FIG S4, TIF file, 1.0 MB. Copyright © 2021 Mohammadzadeh et al. 2021 Mohammadzadeh et al. https://creativecommons.org/licenses/by/4.0/ This content is distributed under the terms of the Creative Commons Attribution 4.0 International license.

### Data availability.

All data are available in the main text or the supplemental materials.
