# Supplementary figures and images for: Lentiviral Infections Persist in Brain despite Effective Antiretroviral Therapy and Neuroimmune Activation
Source: mBio. 2021 Dec 14;12(6):e02784-21. doi: 10.1128/mBio.02784-21 (PMC8669467; doi:10.1128/mBio.02784-21)

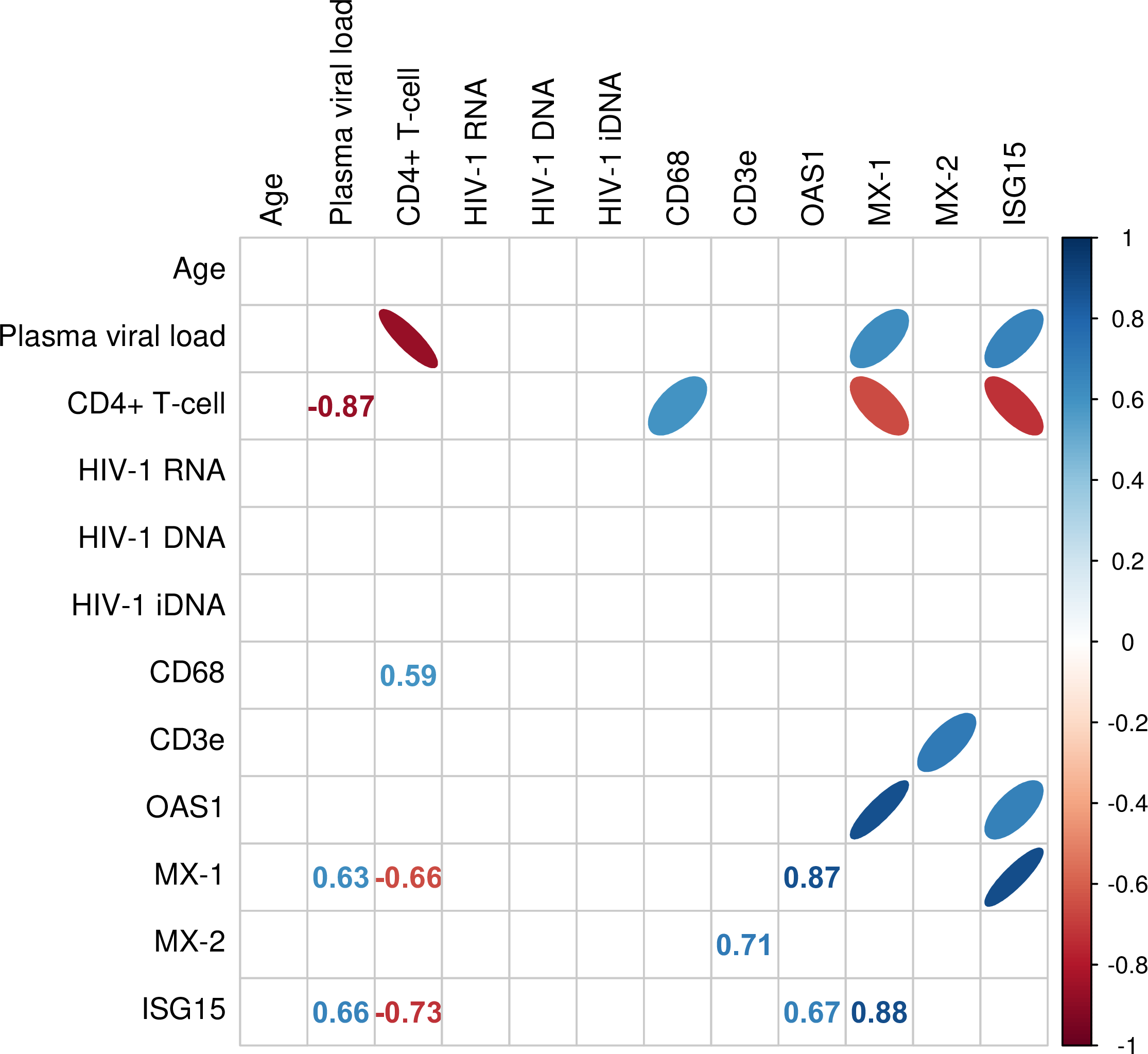

Supplement: FIG S1 [file mbio.02784-21-sf001.tif]

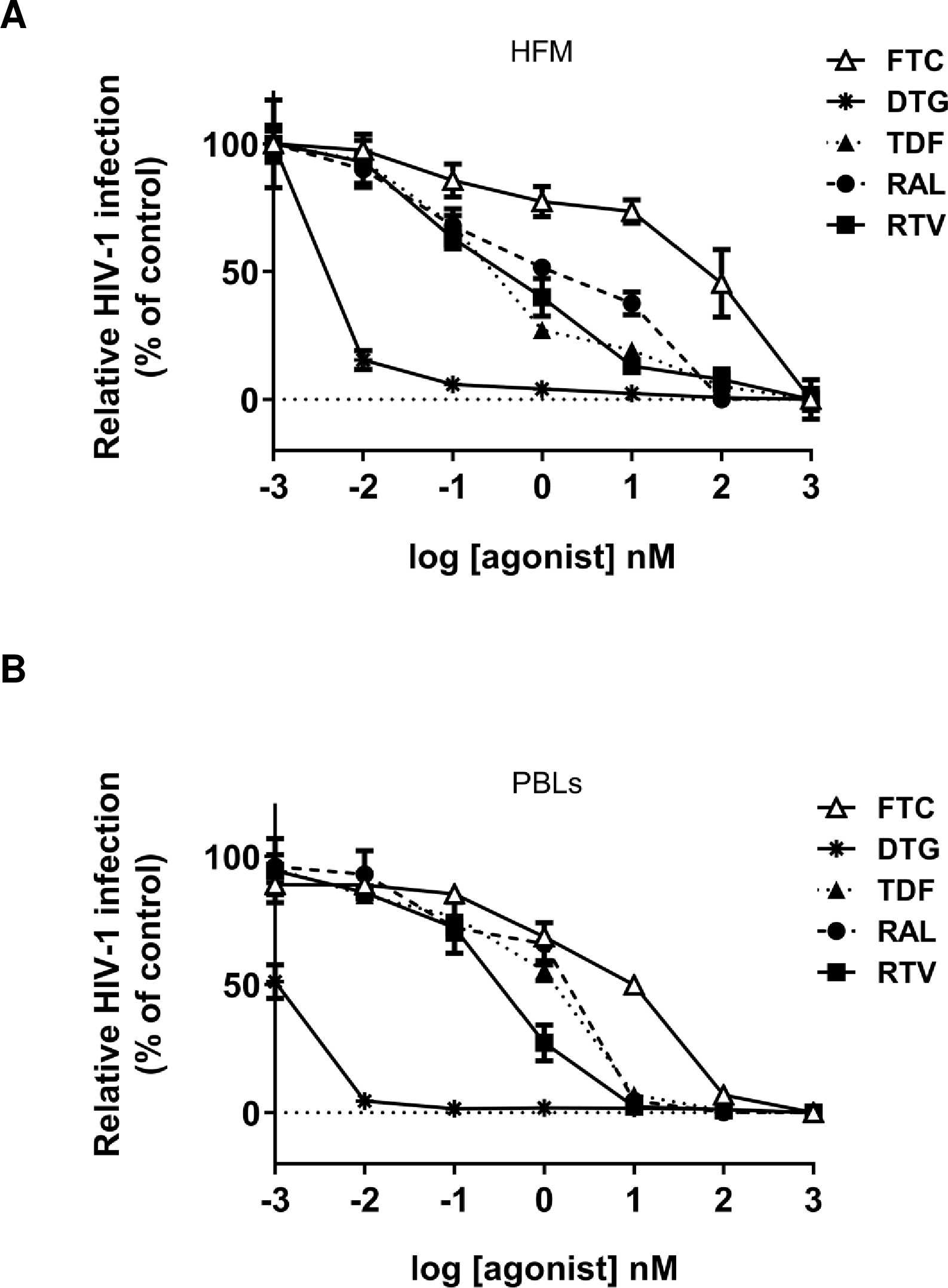

Supplement: FIG S2 [file mbio.02784-21-sf002.tif]

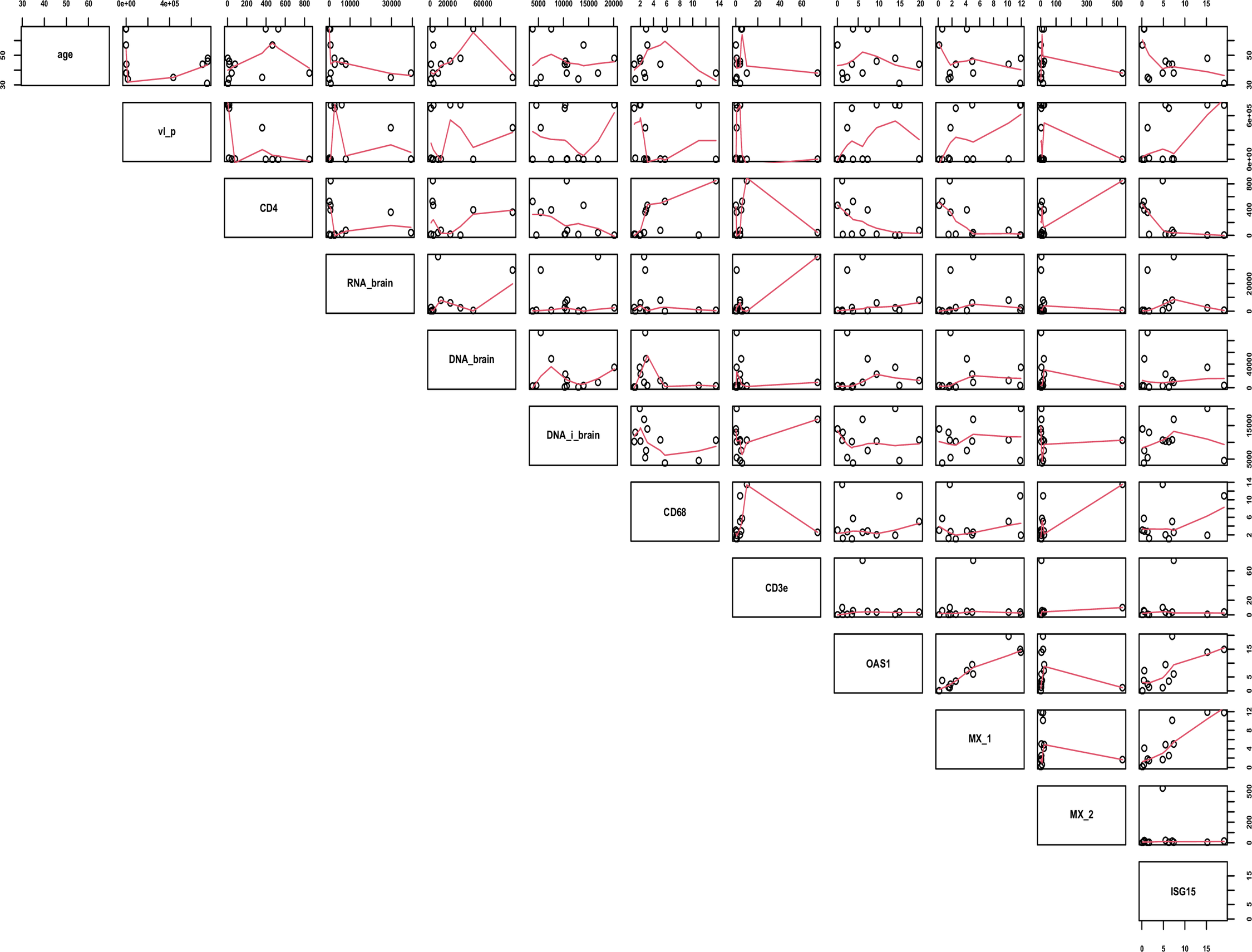

Supplement: FIG S3 [file mbio.02784-21-sf003.tif]

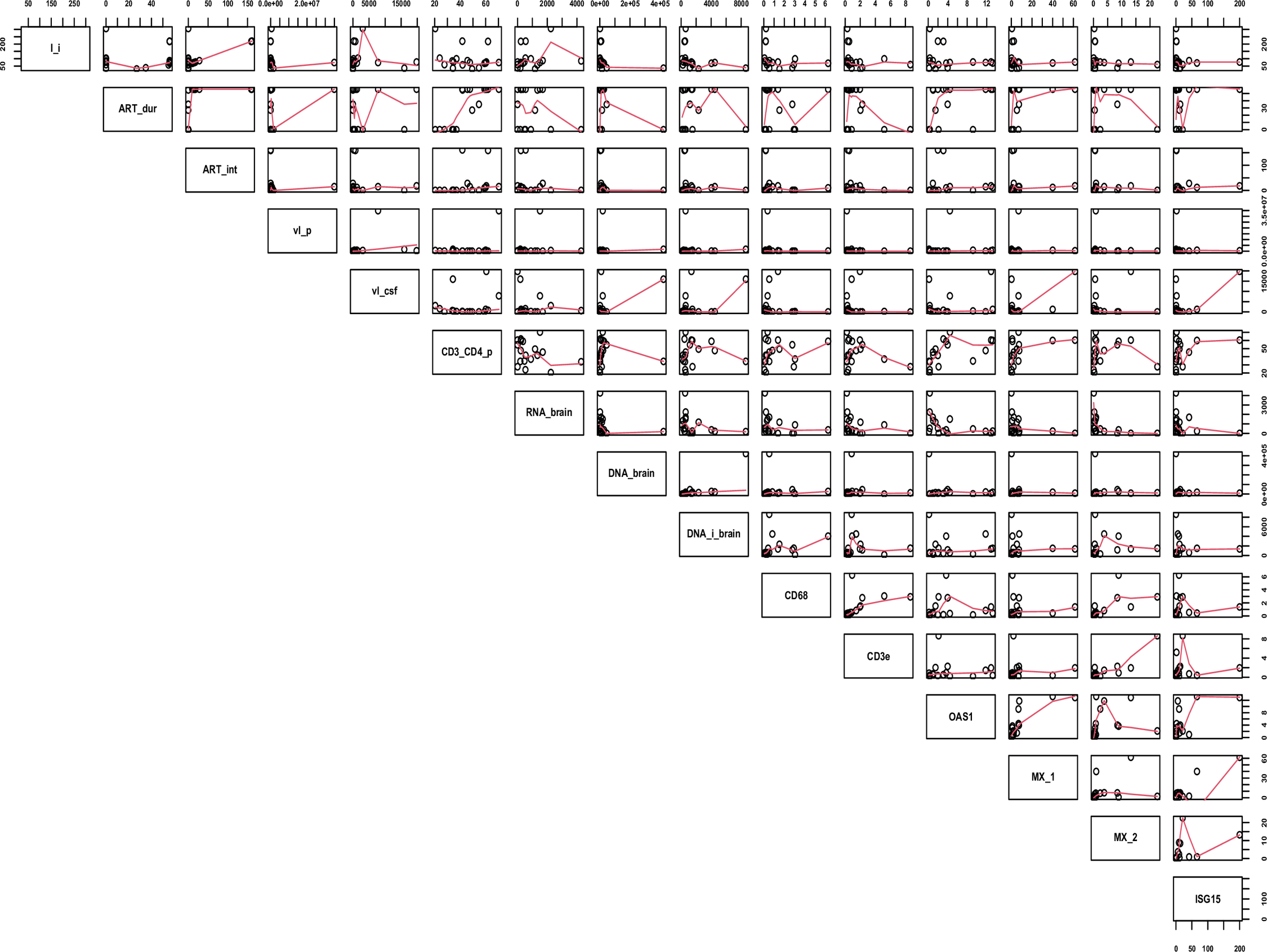

Supplement: FIG S4 [file mbio.02784-21-sf004.tif]
