# Supplementary material for: Lentiviral Infections Persist in Brain despite Effective Antiretroviral Therapy and Neuroimmune Activation
Source: mBio. 2021 Dec 14;12(6):e02784-21. doi: 10.1128/mBio.02784-21 (PMC8669467; doi:10.1128/mBio.02784-21)
Supplement: TABLE S1 [file mbio.02784-21-st001.docx]

**Table S1.** Oligonucleotide primers used for quantitative real time PCR.

| **Human primers:** | |
| --- | --- |
| GAPDH-F | ACCAGGGCTGCTTTTAACTG |
| GAPDH-R | TTGATTTTGGAGGGATCTCG |
| CD68-F | CATCTCTGTACTGAACCCCAAC |
| CD68-R | CCATGTAGCTCAGGTAGACAAC |
| CD3E-F | GCCTCCGCCATCTTAGTAAAG |
| CD3E-R | TCTTCATTACCATCTTGCCCC |
| MX1-F | GAAGATAAGTGGAGAGGCAAGG |
| MX1-R | CTCCAGGGTGATTAGCTCATG |
| MX2-F | CCAGAGGCAGCGGAATCGTAACC |
| MX2-R | GAGCCTTGATCTGCAGTCCGATG |
| ISG15-F | CACAGCCATGGGCTGGGACCTG |
| ISG15-R | CCGGGGCCCAGGCCCTGGCTGGC |
| OAS1-F | ATGATGGATCTCAGAAATACCC |
| OAS1-R | TCAGGAACCCACAGATGA |
| **Chinese rhesus macaque primers:** | |
| GAPDH-F | ACCACCACGGAGAAGGCTGG |
| GAPDH-R | CTCAGTGTAGCCCAGGATGC |
| CD68-F | CATCTCTGTACTGAACCCCAAC |
| CD68-R | CCATGTAGCTCAGGTAGACAAC |
| CD3E-F | GCCTCCGCCATCTTAGTAAAG |
| CD3E-R | TCTTCATTACCATCTTGCCCC |
| MX1-F | ACAGAACCGCCAAGTCCAAA |
| MX1-R | AATGCACCCCTGTACACCTG |
| MX2-F | GCTCCGGAAGCTCCCAC |
| MX2-R | TCTTCCTGACAAGCTCTCTGC |
| ISG15-F | GGCAGTTGAGAGGCAGTGAA |
| ISG15-R | CTTCAGGTCCCAGCTCATGG |
| OAS1-F | ATGATGGATCTCAGAAATACCC |
| OAS1-R | TCAGGAACCCACAGATGA |
