# Supplementary material for: Lentiviral Infections Persist in Brain despite Effective Antiretroviral Therapy and Neuroimmune Activation
Source: mBio. 2021 Dec 14;12(6):e02784-21. doi: 10.1128/mBio.02784-21 (PMC8669467; doi:10.1128/mBio.02784-21)
Supplement: TABLE S2 [file mbio.02784-21-st002.docx]

**Table S2.** Oligonucleotide primers for ddPCR.

| **Human primers:** | |
| --- | --- |
| HIV-1 pol-F | TTAAGACAGCAGTACAAATGGGCAG |
| HIV-1 pol-R | ACTGCCCCTTCACCTTTCCA |
| Alu-F | GCCTCCCAAAGTGCTGGGATTACAG |
| HIV-1 gag-R | GTT CCTGCTATGTCACTT CC |
| **Chinese rhesus macaque primers:** | |
| SIV-F | GCAGAGGAGGAAATTACCCAGTAC |
| SIV-R | CAATTTTACCCAGGCATTTAATGT |
| Alu-F | TCTGCGTCATCTGGTGCATTCACG |
| SIV gag-R | TGCCAACAGGCTCAGAAAATT |
